# Evaluation of Postgraduates Following Implementation of a Focus Assessed Transthoracic Echocardiography (FATE) Training Course-A Pilot Study

**DOI:** 10.4172/2155-6148.1000763

**Published:** 2017-09-27

**Authors:** Stephen C Haskins, Jinhui Zhao, Jemiel A Nejim, Kara Fields, Sean Garvin, Sumudu Dehipawala, James D Beckman, Angie Zhang, James A Osorio, Christopher Tanaka

**Affiliations:** 1 Hospital for Special Surgery, New York, USA; 2 Beth Israel Deaconess Medical Center, Boston, Massachusetts, USA; 3 Johns Hopkins University, Baltimore, Maryland, USA; 4 Weill Cornell Medical College, New York, USA; 5 Montefiore Medical Center, Bronx, New York, USA

**Keywords:** Point-of-Care ultrasound, Focused cardiac ultrasound, Education, Anesthesia trainees

## Abstract

At our institution, implementation of a formal training course in Basic Focus Assessed Transthoracic Echocardiography (FATE) was associated with an improvement in anesthesia trainees’ ability to obtain transthoracic echocardiography (TTE) images. Total image acquisition scores improved by a median (Q1, Q3) 9.1 (2.9,14.7) percentage points from pre-to post-hands-on FATE course (n=20; p=0.001). Participants who returned for a subsequent assessment 5 months following the course demonstrated a median (Q1, Q3) 18.0 (9.1,22.1) percentage point improvement from their pre-course total image acquisition scores (n=11; p=0.002). This pilot study established the feasibility of our program and results suggest that the basic FATE course can be used to teach trainees TTE quickly, effectively, and with significant retention.

## Introduction

As medical technology continues to improve, the paradigm in both intra-operative and peri-operative management shifts from invasive procedures, such as cardiac catheterization and transesophageal echocardiography (TEE), to non-invasive imaging techniques that allow real-time monitoring of the patient’s hemodynamic status. One such technique is the focused transthoracic echocardiography (TTE), which has been demonstrated to be an effective tool for cardiopulmonary monitoring and resuscitation, both peri-operatively and in the intensive care unit [1–3]. Focused TTE in the peri-operative setting can detect significant cardiac pathology and result in a change in management and anesthetic or surgical plan [1,4]. Compared to TEE, TTE also has the added benefits of being non-invasive, rapid and does not require sedation or lengthy cleaning procedures [4].

In addition to its benefits as a diagnostic tool, focused TTE can be taught successfully in a short period of time to physicians without formal training in echocardiography or cardiology [2,5–7]. Despite the growing awareness among anesthesiologists of the benefits of focused TTE, few courses are available and there is a lack of existing standardized curricula to adequately train resident and fellow physicians [2,6,8]. There is also limited understanding on whether, when taught this tool, there is adequate retention of the skill after a prolonged follow-up period.

Currently, several focused TTE protocols exist for the assessment of the heart to evaluate the hemodynamic state of the patient, such as the Ultrasound Hypotensive Protocol (UHP) [9,10], Haemodynamic Echo Assessment in Real Time (HEARTscan) [9], and Focus Assessed Transthoracic Echo (FATE) [3]. FATE provides a rapid and thorough examination of the heart, pericardial space, inferior vena cava, and adjacent pleura [3,11]. The purpose of this study is to assess the feasibility and effectiveness of an online FATE learning module (E-learning) along with a full day hands-on FATE training course for anesthesiology residents and fellows from Hospital of Special Surgery (HSS) and Weill Cornell Medical College (WCMC).

## Consent for Publication

The study was approved by the institutional review board (IRB) at Hospital for Special Surgery (#2014-209). Written informed consent was obtained from all subjects on the day of the hands-on-training course. Inclusion criteria was a CA-2, CA-3 or anesthesiology fellows from either WCMC or HSS taking the Basic FATE training course. Exclusion criteria was refusal to participate in the study.

## Methods and Results Description

**Figure 1:**
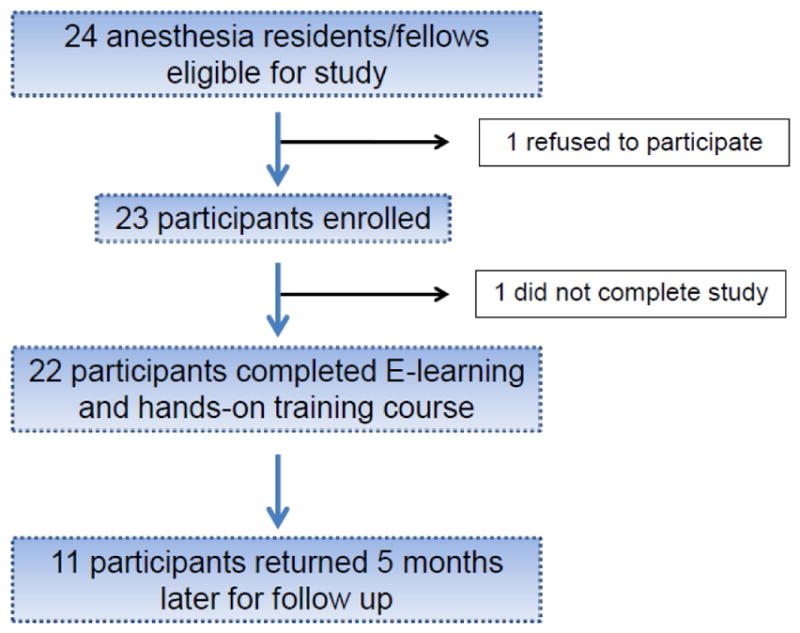
Participant Flow Diagram.

After HSS Institutional Review Board approval (protocol #2014-209, approved September 2014), 23 anesthesiology residents and fellows were enrolled in a prospective cohort study, which took place between October 2014 to March 2015 (Figure 1).

Participants completed a survey prior to the E-learning and handson course regarding their baseline familiarity with ultrasound (Table 1). Participants then completed a didactic online E-learning module on FATE (Basic FATE course, available at http://www.usabcd.org). The E-learning taught participants the following information: utilization of the ultrasound machine knobs to optomize imaging “knobology,” the basic FATE protocol views, relevant anatomy, determination of cardiac dimensions and ventricular function from acquired images and recognition of cardiac pathology. To gauge improvement in knowledge and understanding of the material, scores from pre-and post-Elearning tests were collected and compared.

Following completion of E-learning, participants gathered for a 6 h hands-on training course. 6 stations were set up for the course to enure a maximum of four participants per station. Each station consisted of a portable ultrasound machine (GE LOGIQ-e ultrasound (GE Healthcare, Wisconsin, USA) utilizing a GE 3S-RS sector transducer or SonoSite X-Porte (FUJI Film SonoSite Inc) utilizing a sonosite p21xp transducer, a healthy male volunteer as the “patient”, and an attending anesthesiologist trained in focused transthoracic echocardiography.

Participants were placed in groups of three or four, and each group rotated between stations throughout the day. To obtain estimates of the central tendency and variability of the pre, post, and change in image acquisition score associated with the hands-on training course, participants were asked to capture ultrasound images of each of the 6 FATE-protocol views before and after the course. There was a time limit of 90 s for each image acquisition. Scoring of the images was based on a validated system that evaluates each image based on five aspects: anatomical presentation, sector optimization (depth and angle width), gain adjustment, image (resolution) sharpness, and interpretational value [5]. The pre- and post- course image acquisition scores were collected and analyzed. Due to technical difficulties experienced during course implementation, some FATE views acquired by 4 participants were deleted from the system before scoring. These images were not included in the final analysis.

5 months after completion of the course, 11 of the 22 trainees returned, reported their level of TEE and TTE exposure in the 5 months follow-up period, performed a basic FATE exam on a healthy volunteer and saved images for scoring at a later time. To minimize potential grading bias, images were scored by two blinded anesthesiologists with expertise in cardiac ultrasound and the total score for each view was used for data analysis. The scores were then compared to pre-course scores and assessed for retention of material and FATE skill. The scores were also used to gauge correlation between participant-reported ultrasound experience and 5 months follow-up scores.

**Figure 2:**
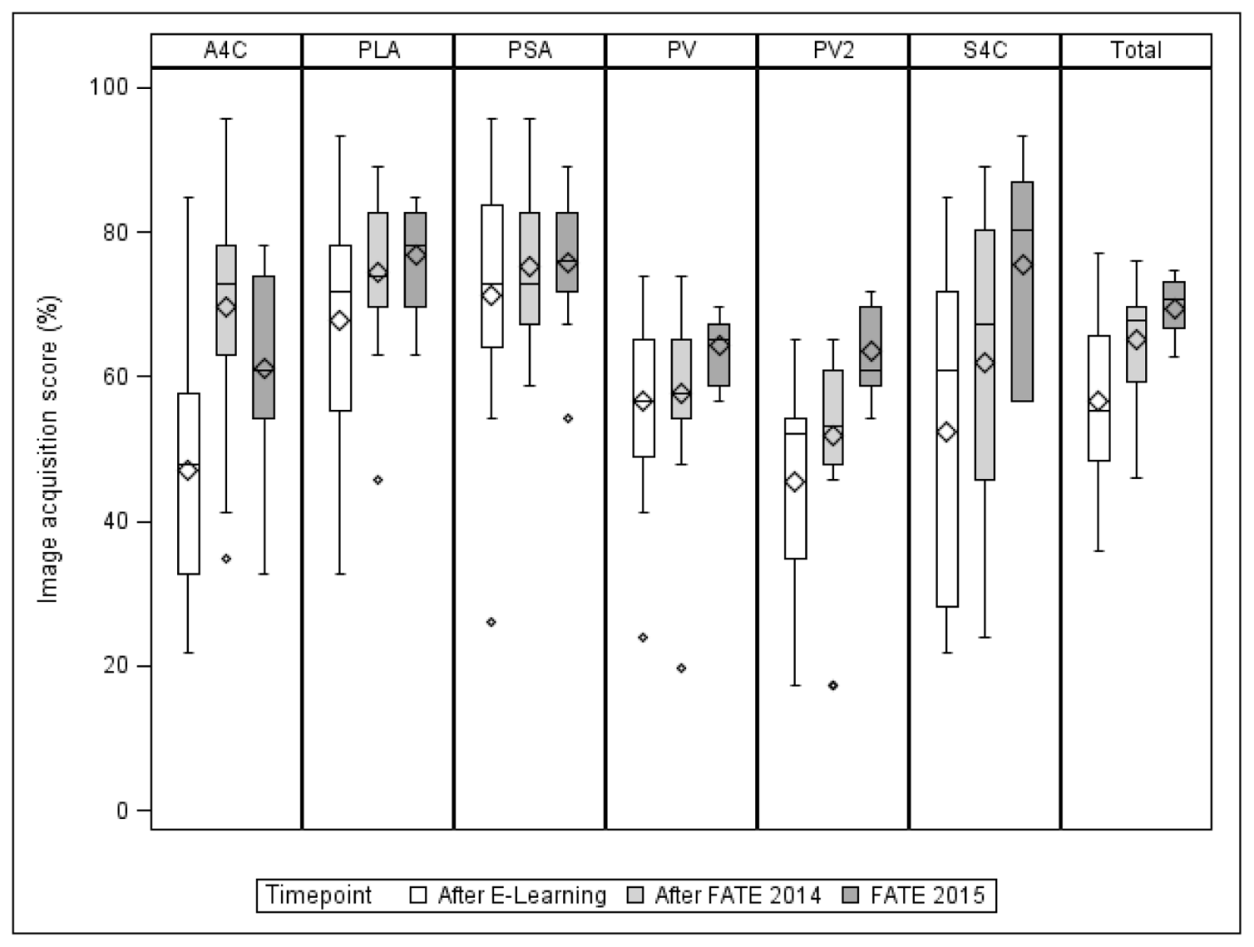
Box-and-whisker plots of image acquisition scores (in percentage points) after completion of online module but before hands-on training course (“After E-learning”), after completion of hands on training course (“After FATE 2014”), and at follow-up 5 months later (“FATE 2015”).

The goal of this study was to obtain estimates of the central tendency and variability of the pre, post and change in image acquisition score associated with the hands-on FATE training course. Therefore, no formal power analysis was performed. Data was collected and managed using Research Electronic Data Capture (REDCap) tools hosted at WCMC. REDCap is a secure, web-based application designed to support data capture for research studies [12]. Discrete variables are presented as counts and percentages, and continuous variables are presented as medians with 1^st^ and 3^rd^ quartiles. The Wilcoxon signed-rank test was used to assess whether changes in online test and image acquisition scores between assessments were different from zero. Results are shown in Figure 2. Correlations between survey responses and image acquisition scores were summarized as Spearman’s correlation coefficients (ρ) with 95% confidence intervals and p values. All statistical hypothesis tests were two-sided, with P<0.05 considered statistically significant. Statistical analyses were performed with SAS Version 9.3 (SAS Institute, Cary, NC).

In our study, E-learning provided the didactic knowledge and was associated with a median (Q1, Q3) 40.4 (22.8,56.3) percentage point increase in comprehension of basic FATE between the online pre-test and post-test (p<0.001). As for the hands-on training course, Figure 2 illustrates image acquisition scores before and after completion of hands-on training course as well as image acquisition scores 5 months after completion of the course. Overall, there was considerable improvement in total image acquisition scores from pre-course to post-course imaging, with improvement in all 6 FATE-protocol views. The greatest improvement was in the apical 4 chamber view, with a median (Q1, Q3) 23.9 (8.7,39.1) percentage point increase (n=19; p=0.001). Of the 11 participants that returned, there was excellent retention 5 months following training. The total score from the acquired images from pre-course to 5-month follow-up improved by a median (Q1, Q3) 18.1 (9.1,22.1) percentage points (n=11; p=0.002). The greatest increase was in the subcostal 4 chamber view, with a median (Q1, Q3) 30.4 (4.3,58.7) percentage point improvement as compared to pre- course scores (n=11; p=0.001). This is especially important given that the subcostal 4 chamber view is a difficult view to master and competency in acquiring this view is critical for comprehensive evaluation of the hemodynamic status of the patient. Table 2 shows correlation of ultrasound experience during the follow-up period and follow-up image acquisition scores. The number of TTEs performed was strongly correlated with overall image acquisition scores at 5 months (ρ=0.61, 95% CI: 0.01–0.89, p=0.046). Furthermore, the strongest correlation was between the number of TTEs performed and 5-month follow-up score for the subcostal 4 chamber view (ρ=0.81, 95% CI: 0.41–0.95, p=0.002).

## Discussion

The 6 views obtained from a FATE exam provide sufficient and reliable information to assist the clinician with pre-operative evaluation, intra-operative monitoring, and post-operative management [2]. In addition to applications in cardiac anesthesia and the intensive care unit, FATE has been applied to evaluate cases of hemodynamic instability in the setting of pleural effusions [11] and shock [13]. Furthermore, FATE has been successfully used to provide adequate diagnostic information in challenging patient populations like post-thoracic surgery patients and patients who cannot tolerate the supine position [2,14].

At our institution, implementation of a formal training course in FATE improved anesthesia trainees’ ability to obtain TTE images suitable for interpretation. There was improvement in ability to perform a FATE exam following the 6-h hands-on training course for participants, most of whom with no previous experience with focused TTE. Additionally, these trainees were able to retain FATE-protocol skill up to 5 months following the initial training, even without extensive post-course practice. This pilot study demonstrates that implementation of a formal training course in Basic FATE to anesthesiology residents and fellows is feasible. Furthermore, data obtained from this study can be used for power analysis in future studies. Lastly, the experience gained by researchers during program implementation is invaluable for curriculum design and planning for subsequent, larger studies involving focused TTE pedagogy.

Given a recent call to action for a formal perioperative ultrasound training for anesthesiology residencies [15], in addition to a push to embrace focused cardiac ultrasound within anesthesia subspecialties [16] as well as the profession in general [17], it is clear that formal training in this skill will need to be embraced by anesthesia training programs in the not to distant future. As the anesthesiologist’s role continues to evolve around peri-operative management and care, the use of point-of-care and goal-directed ultrasonography should be incorporated into daily practice to help guide fast and accurate diagnoses of potentially life-threatening conditions in time-limited situations. As such, residency programs can organize short yet effective training sessions such as the one reported to close this professional practice and educational training gap.

## Figures and Tables

**Table 1:** Baseline participant information.

		Returned for assessment of skill 5 months after course		All
		No	Yes	23
All		12	11	
Level of Training	Resident CA-2	2 (16.70%)	1 (9.10%)	3 (13.00%)
	Resident CA-3	4 (33.30%)	4 (36.40%)	8 (34.80%)
	Fellow-Cardiac	2 (16.70%)	1 (9.10%)	3 (13.00%)
	Fellow-Regional	3 (25.00%)	5 (45.50%)	8 (34.80%)
	Fellow-Obstetric	1 (8.30%)	0	1 (4.30%)
Rank your experience with Ultrasound (all forms including peripheral nerve blocks and vascular access)	very limited (less than 20 times)	1 (8.30%)	0	1 (4.30%)
	somewhat (between 20–50 times)	4 (33.30%)	5 (45.50%)	9 (39.10%)
	very (between 50–100 times)	3 (25.00%)	4 (36.40%)	7 (30.40%)
	extensive (>100 times)	4 (33.30%)	2 (18.20%)	6 (26.10%)
Have you ever performed a focused TTE?	No	11 (91.70%)	8 (72.70%)	19 (82.60%)
	Yes	1 (8.30%)	3 (27.30%)	4 (17.40%)
How familiar are you with focused TTE?	Aware but never seen or used	1 (8.30%)	2 (18.20%)	3 (13.00%)
	Seen but never performed	9 (75.00%)	7 (63.60%)	16 (69.60%)
	Performed limited number, but no formal training	2 (16.70%)	2 (18.20%)	4 (17.40%)

**Table 2:** Correlation between number of cardiac ultrasound exams performed in 5 months the follow-up period and post-course to 5- month follow up change in image acquisition score.

FATE-protocol view		Spearman Correlation Coefficient	95% Confidence Interval		P Value
Overall score	Total	0.46	−0.2	0.83	0.163
	TTE exams	0.61	0.01	0.89	0.046 *
S4C	Total	0.63	0.05	0.89	0.036 *
	TTE exams	0.81	0.41	0.95	0.002 *
A4C	Total	−0.17	−0.7	0.48	0.623
	TTE exams	−0.12	−0.67	0.52	0.733
PLA	Total	0.36	−0.31	0.79	0.287
	TTE exams	0.4	−0.27	0.8	0.237
PSA	Total	−0.53	−0.86	0.11	0.097
	TTE exams	−0.27	−0.75	0.39	0.425
PV	Total	−0.09	−0.65	0.54	0.8
	TTE exams	0.12	−0.51	0.67	0.723
PV2	Total	0.35	−0.31	0.79	0.298
	TTE exams	0.15	−0.49	0.69	0.669

* Statistically significant (p<0.05)

Abbreviations: S4C: Subcostal 4 Chamber view; A4C: Apical 4 Chamber view; PLA: Parasternal Long Axis view; PSA: Parasternal Left Ventricular Short Axis view; PV: Pleural (Left) view; PV2: pleural (Right) view.
